# Targeted redox-responsive peptide for arterial chemoembolization therapy of orthotropic hepatocellular carcinoma

**DOI:** 10.1007/s00261-024-04481-8

**Published:** 2024-07-11

**Authors:** Yimao Xia, Xin Li, Fengyong Liu

**Affiliations:** 1 Chinese PLA Medical School, Beijing, 100853 China; 2 Department of Interventional Radiology, The Fifth Medical Center of PLA General Hospital, Beijing, 100039 China

**Keywords:** Drug delivery, Hepatocellular carcinoma, Peptide, Transcatheter arterial chemoembolization

## Abstract

**Objective:**

Transcatheter Arterial Chemoembolization (TACE) is the first choice for the treatment of advanced-stage hepatocellular carcinoma (HCC). However, TACE suffers from a lack of specificity and rapid drug release. Herein, a targeted redox-responsive peptide (TRRP) was synthesized and used as a carrier of doxorubicin (DOX) to enhance the efficacy of TACE through tumor cells targeting and controlled drug release.

**Methods:**

TRRP has a high loading capacity of DOX and a sensitive drug release behavior at high glutathione (GSH) concentration. Moreover, TRRP could bind to the transferrin receptor on the surface of tumor cells, which enhanced the efficacy of TACE and reduced side effects of TACE. TACE with TRRP@DOX dispersed in lipiodol shows an enhanced therapeutic outcome compared to the treatment with DOX + lipiodol emulsion in orthotopic rat HCC models.

**Results:**

TRRP has a high loading capacity of DOX and a sensitive drug release behavior at GSH concentration. Moreover, TRRP could bind to the transferrin receptor on the surface of tumor cells, which enhanced the efficacy of TACE and reduced side effects of TACE. TACE with TRRP@DOX dispersed in lipiodol shows an enhanced therapeutic outcome compared to the treatment with DOX + lipiodol emulsion in orthotopic rat HCC models.

**Conclusions:**

This study demonstrated that TRRP was a promising therapeutic agent for enhancing TACE therapy for HCC treatment.

**Graphical abstract:**

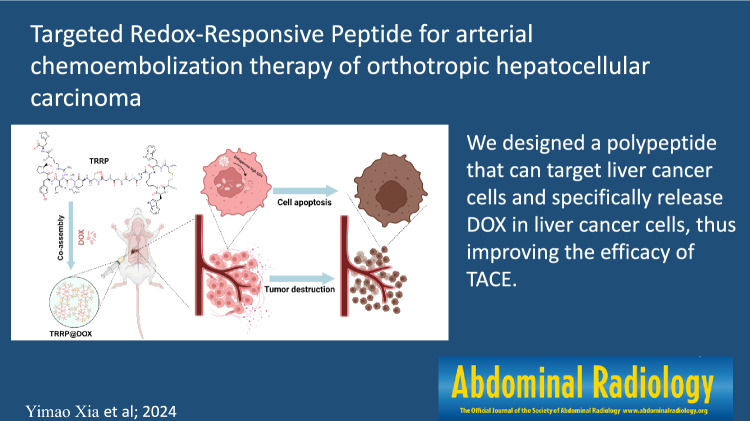

**Supplementary Information:**

The online version contains supplementary material available at 10.1007/s00261-024-04481-8.

## Introduction

Hepatocellular carcinoma (HCC) is a major concern worldwide, ranking fifth in new cases and third in mortality rates [1–3]. It often goes undetected until later stages, with fewer than 20% of patients suitable for surgical removal [4–6]. Although systemic chemotherapy is an option, it hasn’t shown substantial benefits for HCC patients and comes with severe side effects [7–9].

Recently, Lipiodol-based transcatheter arterial chemoembolization (TACE) has become the main palliative treatment for inoperable HCC, showing significant effectiveness in intermediate and advanced cases [10, 11]. However, TACE has limitations. Firstly, its lack of specificity raises concerns as it may harm healthy liver tissues [12, 13]. Secondly, there are challenges in controlling drug release within the tumor site using both natural and external triggers [14, 15]. Thus, there’s an urgent need for an innovative drug delivery system to improve TACE’s efficacy. Incorporating multifunctional nanomaterials into TACE shows promise in overcoming these obstacles [16–18]. These nanomaterials can carry drugs, target specific sites, and release drugs in response to stimuli, making them suitable for TACE [19, 20]. However, their preparation process is often complex and their toxicity raises concerns [21–24]. Peptides, due to their safety profile, stand out as potential carriers for drugs. They can be synthesized quickly, controlled precisely, and enhanced with specific ligands for targeted delivery, minimizing risks to non-target cells and facilitating drug elimination [25–28]. The transferrin receptor (TfR) is crucial in cellular iron uptake, overexpressed on liver cancer cells’ surface, making it a potential target for drug delivery [29–32]. Additionally, liver cancer cells have high intracellular glutathione levels, suggesting potential targeting opportunities [33, 34].

To address the challenges in TACE, we propose an innovative approach: the synthesis of reductively responsive self-assembled peptides targeting hepatoma cells using solid-phase synthesis. This strategy, named Targeted Redox-Responsive Peptide (TRRP), selectively targets overexpressed transferrin receptors on liver cancer cell surfaces [35–37]. Additionally, it possesses redox-responsive characteristics, enabling drug release within tumor cells when exposed to elevated levels of glutathione [38, 39]. Moreover, it demonstrates exceptional biocompatibility [40]. We thoroughly examined TRRP’s physicochemical properties using spectroscopy and electron microscopy analysis. Rat McA-RH7777 liver tumor models, resembling human HCC, were established, and DOX-loaded TRRP (TRRP@DOX) was administered through the hepatic artery in TACE. Through blood biochemistry analysis, dynamic MRI, and endpoint pathological and immunohistochemical evaluations, our research suggests that peptides enhance TACE’s effectiveness in liver cancer treatment while reducing adverse effects. This presents a novel and promising treatment approach for clinical HCC therapy.

## Materials and methods

### In vitro cell studies

HepG2 (human liver cancer cell line) and LO-2 (human normal hepatic cell line) were procured from Type culture Collection of the Chinese Academy of sciences (Shanghai, China). Cells were cultured in Dulbecco’s modified Eagle’s medium (DMEM) supplemented with 10% fetal bovine serum and 1% penicillin–streptomycin at 37 °C, 5% CO_2_.

### In vitro cell uptake assay

HepG2 and LO-2 cells, grown in 96-well plates (1 × 10^5^ cells/well), were cultured for 24 h. After aspirating the medium, 100 μL of fresh medium containing free DOX, TRRP@DOX, or TRRP was added to each well, with DMEM as control. DOX concentration was 15 μg/mL in both free DOX and TRRP@DOX solutions, while TRRP concentration was 100 μg/mL. After 3 h, the medium was removed, and cells were washed with PBS. Cells were fixed with 4% formaldehyde for 30 min, stained with DAPI for 5 min, washed with PBS, and observed under a fluorescent microscope.

HepG2 and LO-2 cells (4 × 10^5^ cells/well) were seeded in sterile 6-well plates and cultured for 24 h. After washing with PBS, cells were treated with TRRP, DOX, or TRRP@DOX (TRRP: 45 µg/mL, DOX: 15 µg/mL) for 2 h. Fluorescence intensity was measured using a flow cytometer with PE channel.

### Cell-viability MTT assay

Cells (5 × 10^4^ cells/well) were seeded in a 96-well plate and incubated for 24 h. They were then treated with varying concentrations of TRRP (0–75 µg), free DOX (0–25 µg), and TRRP@DOX (DOX concentrations ranging from 0 to 25 µg) for 12 h. Subsequently, cells were exposed to 5 mg/mL MTT solution and incubated for 4 h at 37 °C in the dark. Afterward, Formazan solution was added, and cells were further incubated for 4 h. Absorbance was measured at 570 nm using a microplate spectrophotometer. Triplicate experiments were performed for cell viability assays.

### In vitro anti-tumor studies

1 × 10^5^ HepG2 cells and LO-2 cells were seeded in a 6-well plate and incubated overnight. Afterward, the media were replaced by fresh complete media with TRRP, free DOX, and TRRP@DOX at an equivalent DOX dose of 15 µg for another 4 h. After being digested and resuspended, the cells were washed twice with PBS, which were then stained with Annexin-V-FITC (5 µL, excited at 488 nm) and 7-AAD (5 µL, excited at 546 nm) for 5 min in the dark. Finally, the degree of cell apoptosis was measured by a flow cytometer (Beckman Coulter, USA).

### Orthotopic HCC rat model

The animal experiment proposal was approved by the board of the Animal Welfare Committee of the Chinese People’s Liberation Army General Hospital (KY-2021-11-23-1). Sprague Dawley (SD) rats weighing 220 ± 20 g were used. To establish the orthotopic McA-RH7777 rat model, 100 µL of McA-RH7777 cell suspension (5 × 10^6^ cells in PBS) was injected into the left lateral lobe of the liver of each rat. Penicillin (2 × 10^5^ units) and dexamethasone (2 mg) were intraperitoneally injected for 3 days post-transplantation to prevent infection and provide immunosuppression.

### In vivo anti-HCC efficacy in HCC-bearing rats

In the McA-RH7777 rat syngeneic orthotopic HCC model intervention, the abdominal cavity was opened to identify and carefully dissect the hepatic and gastroduodenal arteries. The gastroduodenal artery was freed and sutures were placed at the proximal and distal ends. Following distal end ligation, a 26G indwelling needle (Jierui, China) was inserted into the gastroduodenal artery and advanced into the hepatic artery under direct vision. Different treatments were administered into the hepatic artery, including Control, Lipiodol, Lipiodol + DOX, and Lipiodol + TRRP@DOX (equivalent to 0.3 mg DOX), with 5 rats per group. After drug administration, the indwelling needles were removed and the near-end was sutured to stop bleeding.

On days 0, 3, and 7, all rats underwent mild anesthesia and were examined using a 3.0-T MR scanner (PharmaScan 70, Bruker). Fast-saturated T2-weighted images were acquired in transverse, with sequence parameters: TR/TE = 3158/33 ms, slice thickness = 1 mm, and matrix = 256 × 256. Tumor volume (V) was estimated using the largest (L) and smallest (S) diameters with the formula (1):

$$V({mm}^{3})=\frac{(L\times {S}^{2})}{2}$$

Tumor growth inhibition (TGI) rate is one of the most commonly used metrics to quantify the drug response of treatment groups compared to the control group [41]. The basic way to calculate TGI is following:

$$TGI=\left(1-\frac{\text{V}\left(\text{T}\right)}{\text{V}\left(\text{C}\right)}\right)\times 100\%$$

where V(T) and V(C) represent the mean tumor volume of treatment groups and the mean tumor volume of control at the same time after operation.

Caudal vein blood from SD rats was collected before and after treatments for 1, 3, and 7 days. Levels of ALT, AST, CREA, and CK were measured using an automatic biochemical analyzer (Toshiba) to assess hepatorenal function and myocardial toxicity.

On day 7, rats were euthanized, and tumor and major organ samples were collected for histological and IHC analysis. Samples were fixed in formalin, embedded in paraffin, and sectioned into 5 µm-thick slices. H&E staining was performed for general histology, while TUNEL and DAPI staining identified apoptotic cells. Anti-CD31 and anti-VEGF staining assessed angiogenesis, and anti-Ki67 staining evaluated tumor proliferation. Slides were analyzed with a TissueFAXS microscope, utilizing integrated optical density (IOD) for quantitative IHC analysis.

### Statistical analysis

Each experiment was repeated three times, and the results were expressed as mean ± standard deviation statistical analysis was performed using the statistical *t*-test method. Data between different groups were compared via a one-way analysis of variance. The following P-values were considered statistically significant: P < 0.05:*, P < 0.01:**, P < 0.001:***.

## Result

### Cellular drug uptake and cytotoxicity of TRRP@DOX

**Fig. 1:**
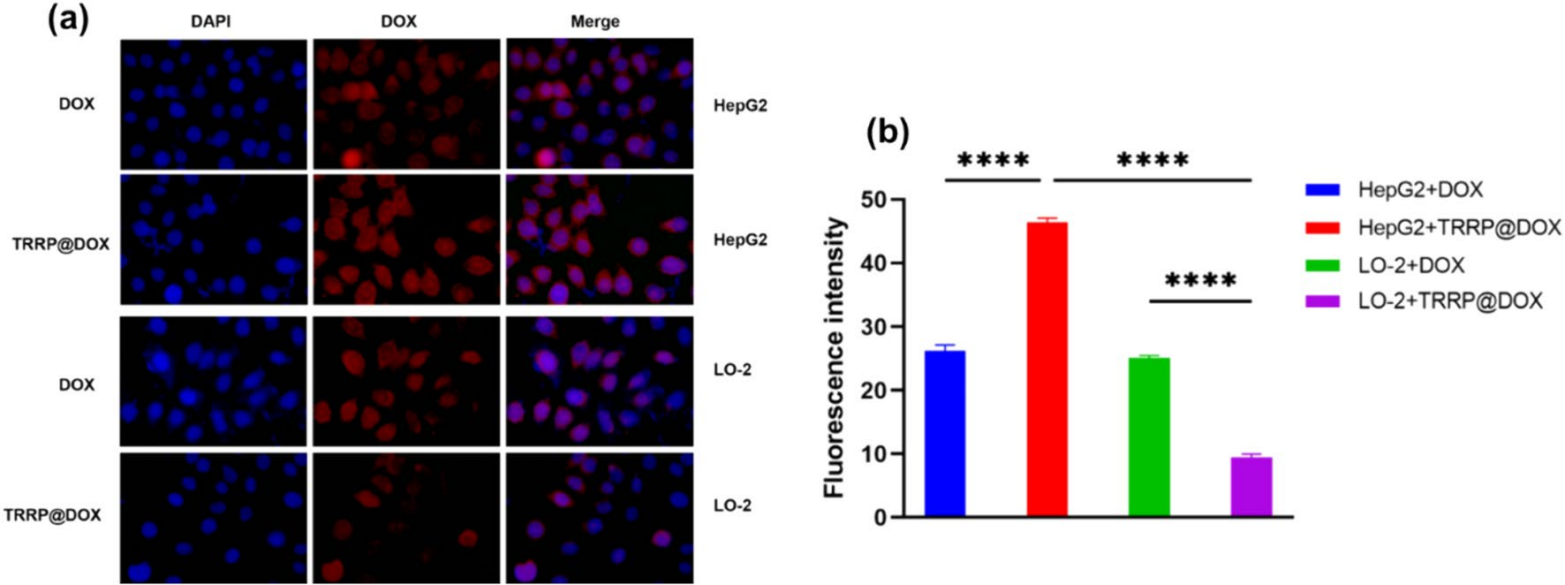
**a** Fluorescence inverted microscope image of HepG2 and LO-2 cells that were incubated with free DOX and TRRP@DOX for 3 h, respectively. Blue and red dots stand for the fluorescence of DAPI and DOX, respectively. All the images were captured at 400 × magnification. **b** Fluorescence intensity of DOX and TRRP@DOX in HepG2 and LO-2 cells after 3 h. ****P < 0.0001

In Fig. 1a, both HepG2 and LO-2 cells exhibited uptake of DOX, evidenced by similar fluorescence intensity. However, TRRP@DOX uptake differed significantly between the two cell types, with lower fluorescence observed in LO-2 cells compared to HepG2 cells, indicating targeted binding to tumor cells. As shown in Fig. 1b, in HepG2 cells, the DOX and TRRP@DOX groups were 26.18 ± 0.95 and 46.43 ± 0.62, respectively, while in LO-2 cells, the DOX and TRRP@DOX groups were 25.13 ± 0.36 and 9.47 ± 0.51, respectively. Statistical analysis revealed no significant difference between the HepG2 + DOX and LO-2 + DOX groups (P = 0.27 > 0.05) Statistical analysis revealed significant differences between groups (*P* < 0.0001).

**Fig. 2:**
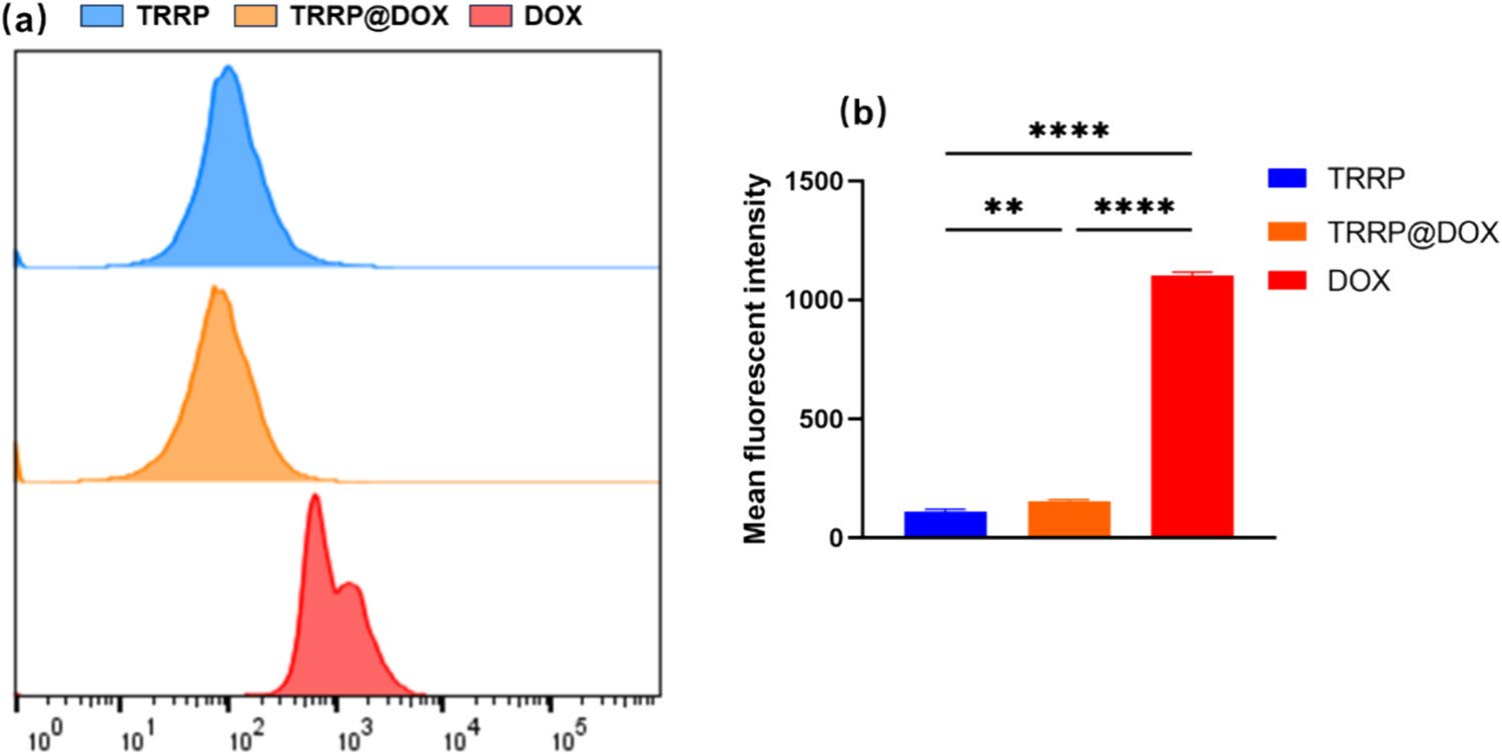
**a** Flow cytometry analysis of TRRP, TRRP@DOX, and DOX uptake by LO-2 cells. **b** Average fluorescence intensity of LO-2 cells after uptake of TRRP, TRRP@DOX, and DOX. **p < 0.01, ****p < 0.0001

PE channel was used to detect the average fluorescence intensity distribution of LO-2 cells for TRRP, TRRP@DOX, and DOX, as shown in Fig. 2a. TRRP@DOX notably reduced DOX uptake by LO-2 cells. Average fluorescence intensity of LO-2 cells co-cultured with TRRP, TRRP@DOX, and DOX was 110 ± 7, 153 ± 5, and 1098 ± 10, respectively (Fig. 2b). Statistical analysis indicated significantly lower fluorescence intensity in LO-2 cells after TRRP@DOX uptake compared to DOX uptake (P < 0.0001), demonstrating the effectiveness of TRRP@DOX in reducing DOX uptake by LO-2 cells.

**Fig. 3:**
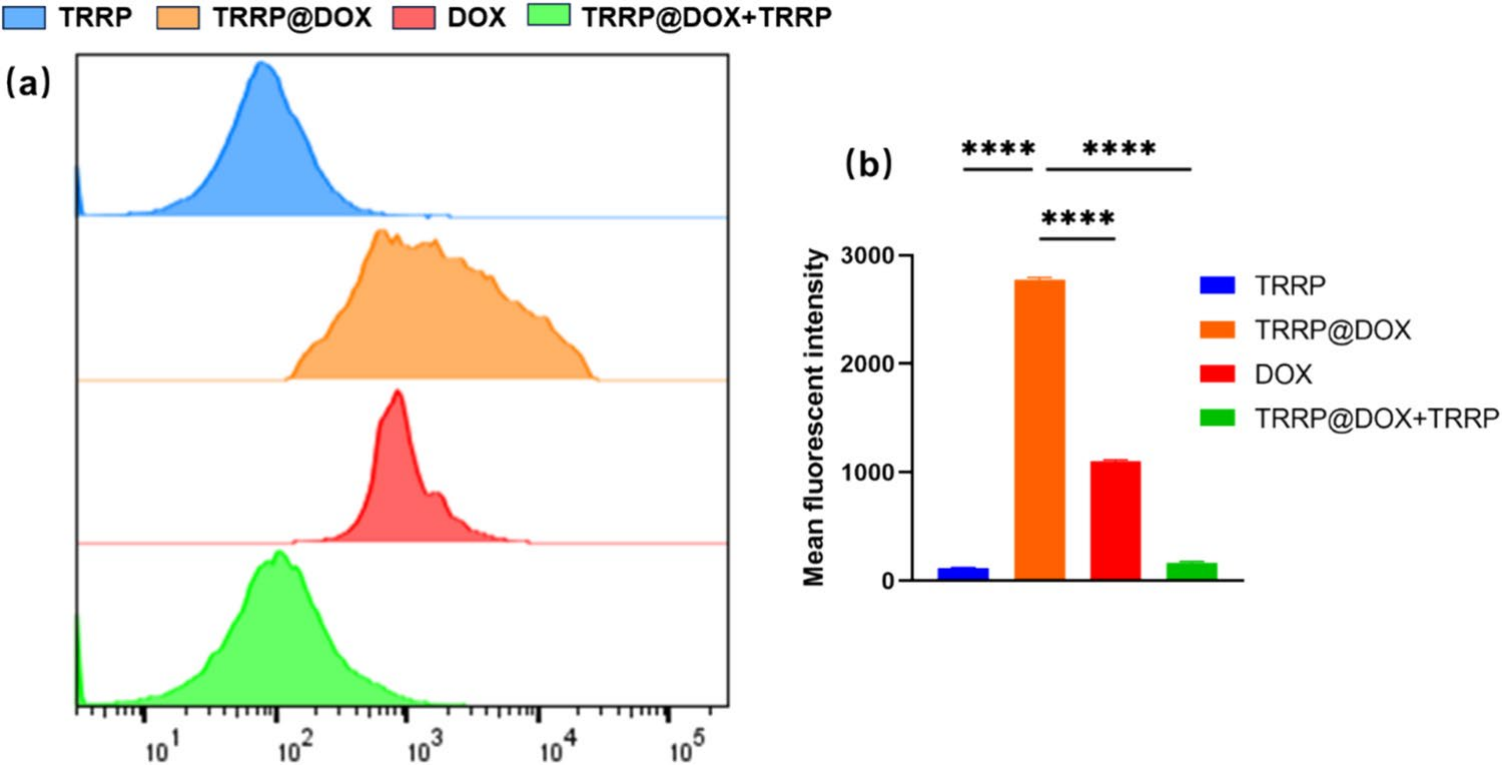
**a** Flow cytometry analysis of TRRP, TRRP@DOX,DOX,and TRRP@DOX + excess TRRP uptake by HepG2 cells. **b** Average fluorescence intensity of LO-2 cells after uptake of TRRP, TRRP@DOX, and DOX. **p < 0.01, ****p < 0.0001

**Fig. 4:**
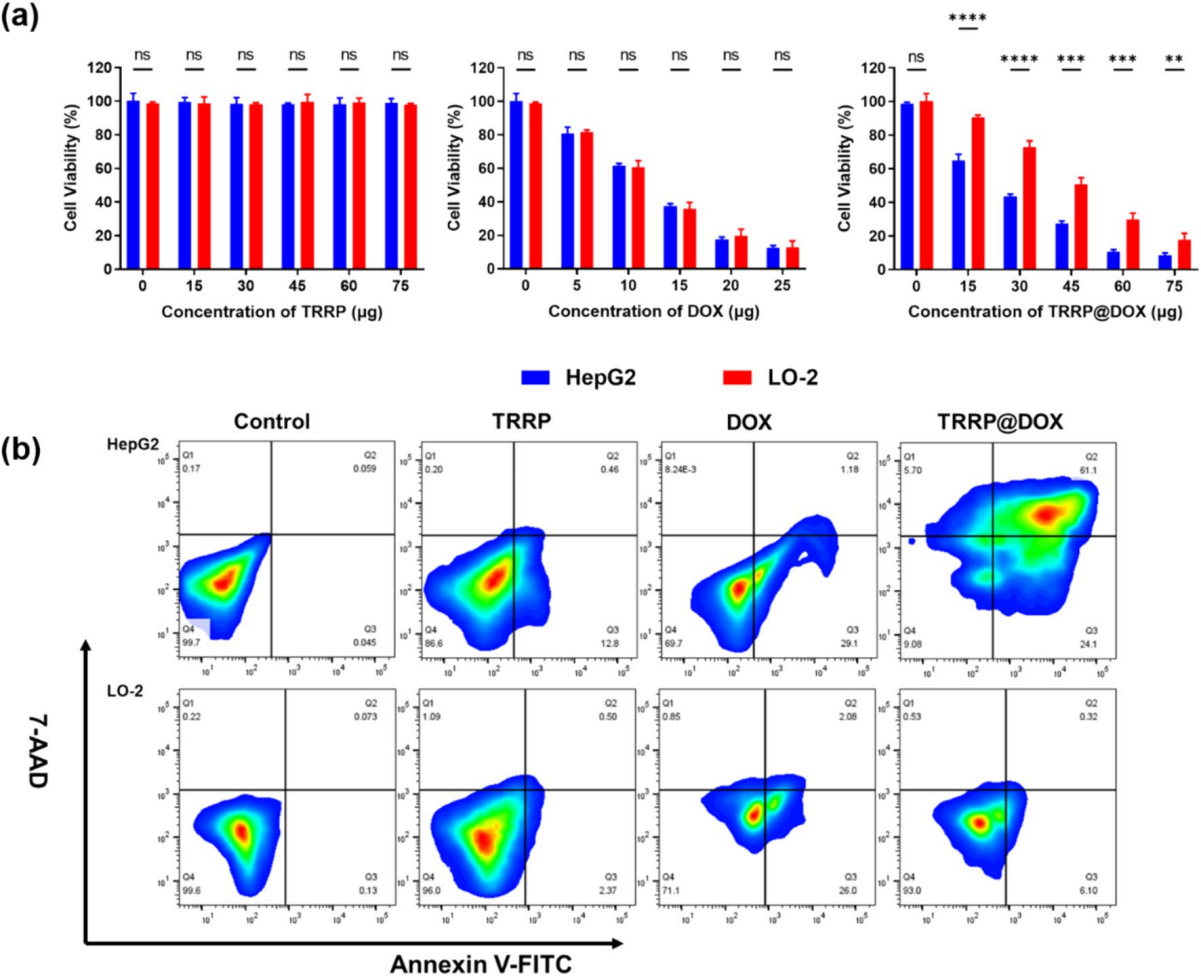
Effects of TRRP@DOX on cell proliferation in vitro **a** Cell viability of HepG2 cells and LO-2 cells after different treatments. **b** Cell apoptosis of HepG2 and LO-2 cells after different treatments. *p < 0.05, **p < 0.01, ***p < 0.001

**Fig. 5:**
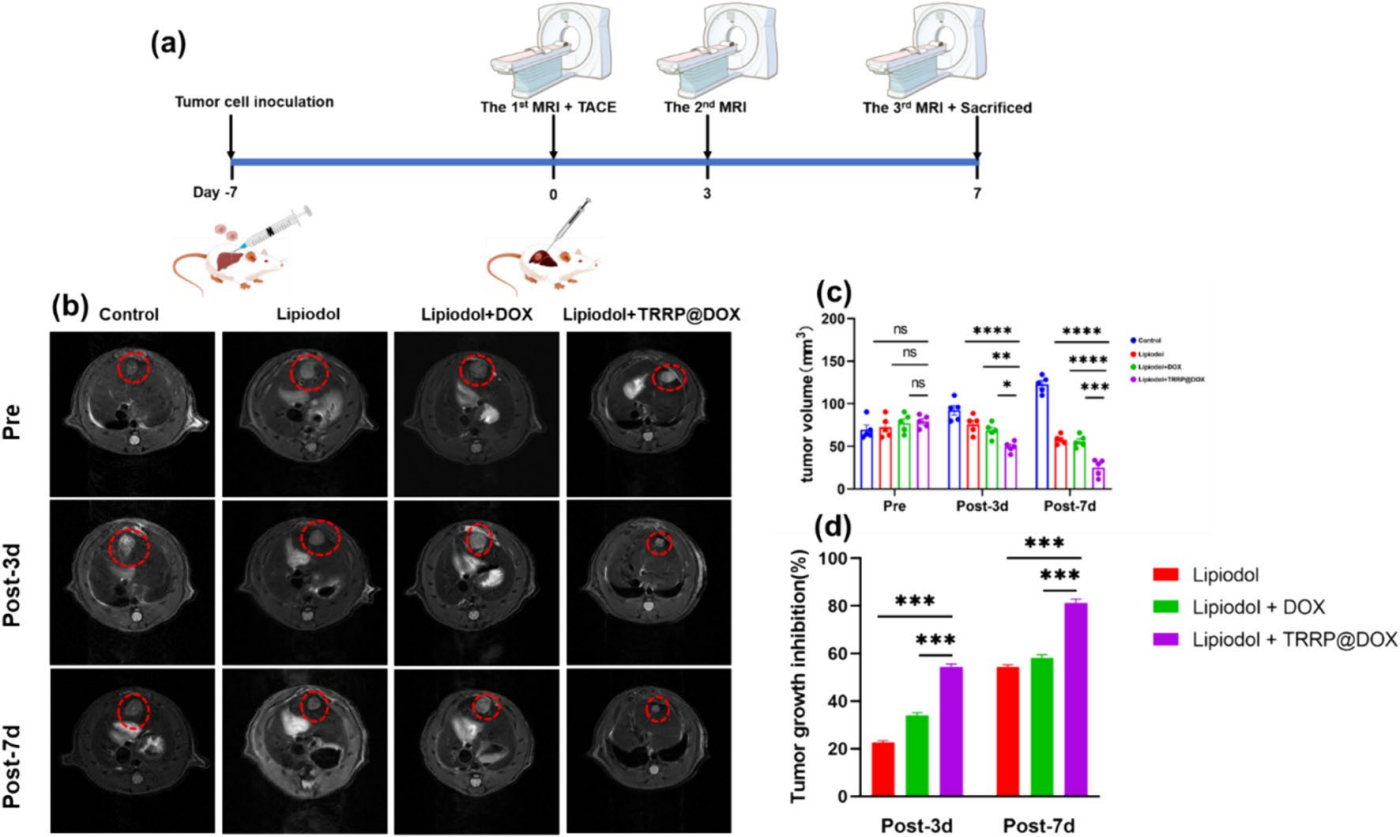
Treatment efficacy of TRRP@DOX in a rat orthotopic HCC model. **a** Schematic of evaluation process. **b** Representative MRI images. **c** Quantitative analysis of relative tumor. **d** Tumor growth inhibition rates were assessed in rats at 3 and 7 days postoperatively. *p < 0.05, **p < 0.01, ***p < 0.001

PE channel was used to detect the average fluorescence intensity distribution of HepG2 cells for TRRP, TRRP@DOX, DOX, and TRRP@DOX + excess TRRP, as shown in Fig. 3a. TRRP@DOX notably increased DOX uptake by HepG2 cells. Additionally, upon adding excess TRRP, we observed a significant reduction in TRRP@DOX uptake by HepG2 cells. This suggests that TRRP competes with TRRP@DOX for TfR binding, thereby reducing TRRP@DOX uptake by HepG2 cells and indirectly demonstrating TRRP’s ability to target HepG2 cell surfaces overexpressing TfR. In Figs. 2, 3, 4, 5b, average fluorescence intensity of HepG2 cells after co-culturing with TRRP, TRRP@DOX, DOX, and TRRP@DOX + TRRP were 115 ± 5, 2785 ± 10, 1096 ± 6, and 168 ± 7, respectively. Statistical analysis showed significant differences between the TRRP@DOX group and the others (P < 0.0001).

MTT assay assessed the impact of TRRP, DOX, and TRRP@DOX on HepG2 and LO-2 cell viability (Fig. 4a). TRRP at concentrations of 0–75 µg/mL showed minimal toxicity after 24 h, indicating its excellent biosafety. Treatment with free DOX (0–25 µg/mL) revealed a dose-dependent decrease in cell viability, suggesting non-selective cellular death. Notably, TRRP@DOX treatment significantly reduced cell viability, particularly in HepG2 cells compared to LO-2 cells. Subsequently, flow cytometric analysis with Annexin V-FITC and 7-AAD staining quantified cell apoptosis (Fig. 4b). Consistently, TRRP@DOX induced stronger apoptosis in HepG2 cells than DOX, while LO-2 cells exhibited lower apoptosis with TRRP@DOX compared to DOX. These results highlight the tumor cell targeting ability of TRRP@DOX. Overall, our cytotoxicity experiments confirm that TRRP@DOX reduces toxicity to normal cells while enhancing cytotoxicity against tumor cells. These findings underscore the in vitro specific cytotoxicity of TRRP@DOX against tumor cells.

### Therapeutic efficacy of TRRP@DOX emulsions in orthotopic rat Mca-RH-7777 syngeneic HCC model

The MRI examination of rats in the four groups is shown in Fig. 5b. T2-weighted imaging revealed uniform slightly high signal intensity with clear boundaries, no obvious satellite foci formation around the tumor, and no evidence of ascites formation. Preoperative tumor volumes for the control, Lipiodol, Lipiodol + DOX, and Lipiodol + TRRP@DOX groups were calculated using formula (1), as shown in Fig. 5c, yielding results of 69.67 ± 12.04 mm^3^, 72.62 ± 12.27 mm^3^, 77.36 ± 11.05 mm^3^, and 79.05 ± 7.35 mm^3^, respectively (p = 0.51 > 0.05). There was no statistical difference in preoperative tumor size among the four groups of rats with liver cancer models. Postoperatively at 3 days, the volumes were 92.48 ± 12.28 mm3, 75.78 ± 10.72 mm^3^, 68.62 ± 8.55 mm^3^, and 49.27 ± 6.05 mm^3^, respectively (p < 0.001), showing statistical differences in tumor size among the four groups at 3 days postoperatively. At 7 days postoperatively, the volumes were 122.48 ± 9.68 mm^3^, 57.89 ± 5.28 mm^3^, 55.89 ± 6.94 mm^3^, and 25.07 ± 9.82 mm^3^, respectively (p < 0.001), indicating statistical differences in tumor size among the four groups at 7 days postoperatively. The tumor growth inhibition rates for the Lipiodol, Lipiodol + DOX, and Lipiodol + TRRP@DOX groups at 3 days postoperatively were 21.67 ± 0.88%, 32.93 ± 1.15%, and 53.37 ± 1.14%, respectively, and at 7 days postoperatively were 54.63 ± 1.03%, 58.80 ± 1.32%, and 81.96 ± 1.64%, respectively. The results of TGI indicate that the Lipiodol + TRRP@DOX group exhibited significantly better efficacy compared to the other groups, demonstrating that TRRP@DOX treatment significantly inhibited tumor growth.

**Fig. 6:**
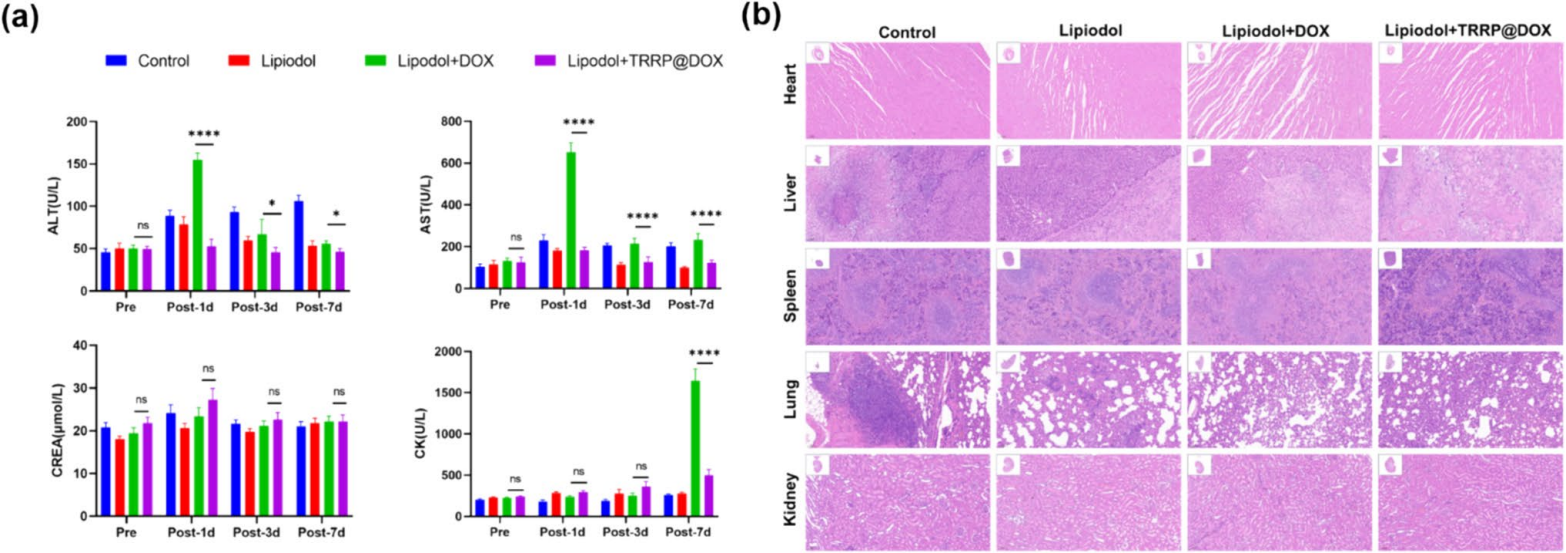
Treatment safety of TRRP@DOX in a rat orthotopic HCC model. **a** ALT, AST, CREA, and CK levels in the blood. **b** H&E staining images of organ in four groups. All the images were captured at 5× magnification (scale bar is 200 µm). *p < 0.05, **p < 0.01, ***p < 0.001

Drug safety is one of the main factors that influence clinical HCC treatment decisions. The toxicity of DOX mainly includes cardiac toxicity, renal toxicity and damage to liver function (Fig. 6a). After embolization, ALT and AST levels of Lipiodol + DOX group increased obviously on day 1 and decreased to normal on day 7, indicating some impairment of liver function. In comparison, the ALT and AST in the lipiodol + DOX group were constantly low and within the normal range from day 1 to day 7, certifying its high safety. Moreover, all the treatment caused negligible damage to the kidney, as supported by normal CREA levels. CK levels of lipiodol + DOX group were higher than those other groups on day 7, indicating myocardial damage. In Lipiodol + TRRP@DOX group, the H&E staining of the heart in this group also proved this point (Fig. 6b). H&E staining of heart, liver, spleen, lung, and kidney tissues showed no significant organ damage on day 7. However, it is noteworthy that metastatic tumors were observed in the lung tissues of all three groups (Control, lipiodol, and lipiodol + DOX); notably, in Lipiodol + TRRP@DOX group, no tumor tissues were observed. This observation underscores the tumor-targeting ability of TRRP@DOX, emphasizing its superior overall efficacy in combating hepatocellular carcinoma. These findings indicate that TRRP@DOX is safe and has a high potential for clinical applications.

**Fig. 7:**
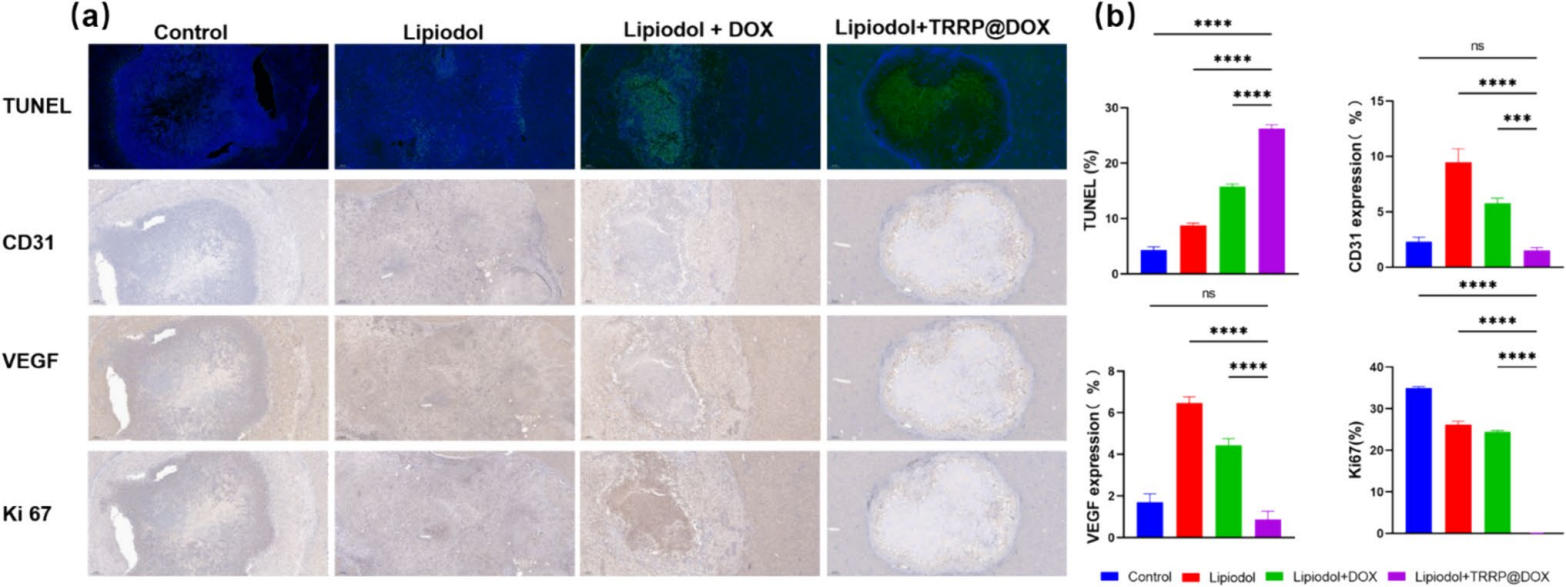
Immunohistochemistry staining of tumor in all treatment groups. **a** Representative TUNEL, CD31, VEGF, and Ki 67 staining of the residual tumor tissues collected from each group to assess the treatment efficacy at the endpoint of the study. All the images were captured at 5× magnification (scale bar is 200 µm). **b** Quantitative immunohistochemical analysis: data were presented as mean ± SD, and analyzed by a one-way ANOVA analysis. *IOD* integrated optical density. *p < 0.05, **p < 0.01, ***p < 0.001

To further evaluate the therapeutic effects, we euthanized the rats with 7 days treatment and collect tumor tissues for histological analysis. H&E staining results showed that the tumor of Lipiodol + TRRP@DOX group had a higher degree of tumor necrosis than other three groups. TUNEL, CD31, VEGF, and Ki 67 immunohistochemical staining further revealed tumor cell apoptosis, angiogenesis, and proliferation, and the representative images are shown in Fig. 7a. The TUNEL-positive rates of tumor tissues in the lipiodol alone group, lipiodol + DOX group, and lipiodol + TRRP@DOX group 7 days postoperatively were 4.33 ± 0.57%, 8.8 ± 0.44%, 15.77 ± 0.46%, and 26.27 ± 0.68%, respectively. Further statistical analysis showed that TRRP@DOX had the highest induction of tumor cell apoptosis (P < 0.0001) compared to the other three groups. The expression rates of CD31 in tumor tissues of the lipiodol alone group, lipiodol + DOX group, and lipiodol + TRRP@DOX group 7 days postoperatively were 2.33 ± 0.40%, 9.46 ± 1.22%, 5.76 ± 0.45%, and 1.53 ± 0.25%, respectively, indicating that TRRP@DOX + lipiodol group had lower tumor microvessel density compared to the lipiodol group (P < 0.0001) and the lipiodol + DOX group (P < 0.001). The expression rates of VEGF in tumor tissues of the lipiodol alone group, lipiodol + DOX group, and lipiodol + TRRP@DOX group 7 days postoperatively were 1.70 ± 0.40%, 6.46 ± 0.32%, 4.43 ± 0.32%, and 0.86 ± 0.40%, respectively, indicating that TRRP@DOX could inhibit neovascularization compared to the lipiodol group (P < 0.0001) and the lipiodol + DOX group (P < 0.0001). The expression rates of Ki 67 in tumor tissues of the lipiodol alone group, lipiodol + DOX group, and lipiodol + TRRP@DOX group 7 days postoperatively were 34.96 ± 0.30%, 26.13 ± 0.83%, 24.43 ± 0.32%, and 0.08 ± 0.04%, respectively, indicating that TRRP@DOX could effectively inhibit tumor proliferation compared to the other three groups (P < 0.0001). Biochemical indicators detected at regular intervals postoperatively suggested that TRRP@DOX did not cause systemic toxic side effects. Pathological examination results 7 days postoperatively showed no tumor metastasis or significant organ damage in the TRRP@DOX group.

## Discussion

In real-world scenarios, liver cancer often eludes early detection, resulting in diagnosis at advanced stages, with fewer than 20% of patients qualifying for surgery [4]. Transarterial chemoembolization (TACE) stands as the preferred treatment for intermediate to advanced liver cancer by delivering a blend of iodized oil and chemotherapeutic drugs directly to tumor-feeding arteries, inducing localized chemotherapy embolization. Despite TACE’s recognized efficacy, challenges persist, notably the systemic release of chemotherapy agents like DOX during injection and embolization, leading to systemic toxicity. Addressing these challenges and enhancing drug targeting in TACE have emerged as crucial pursuits in liver cancer treatment.

TRRP, synthesized in this study, forms disulfide bonds via cysteine residues, facilitating controlled drug release under high glutathione (GSH) concentrations found in tumor cells. While TRRP@DOX releases less than 10% of drugs within 24 h under plasma and normal cell GSH levels, it reaches 65% release within 24 h under tumor cell-equivalent GSH concentrations. Fluorescence microscopy and flow cytometry confirmed specific uptake of TRRP@DOX by HepG2 cells compared to LO-2 cells. Moreover, excess TRRP competitively reduced TRRP@DOX uptake, highlighting the role of TRRP-TfR binding in specific cell uptake. This indirectly proves that TRRP@DOX is specifically uptaked by tumor cells through TfR.

Both MTT assay and flow cytometry demonstrated no cytotoxicity from TRRP, consistent with expectations, affirming TRRP’s suitability as a drug carrier. Subsequent experiments validated TRRP@DOX’s targeted killing of tumor cells while minimizing toxicity to normal cells like LO-2 cells. The targeted uptake of TRRP@DOX by liver cancer cells underscores its efficacy. Notably, tumor cells exhibit higher GSH concentrations than normal cells, enabling controlled drug release from TRRP@DOX within tumor cells’ specific intracellular environments.

In rat liver cancer models, hepatic artery administration of iodized oil and TRRP@DOX yielded tumor growth inhibition rates of 53.37 and 81.96% after 3 and 7 days post-operation, respectively. In contrast, hepatic artery injection of iodized oil and DOX resulted in lower inhibition rates of 32.93 and 58.80%, respectively, underscoring the superior efficacy of TRRP@DOX. Generally, a tumor inhibition rate exceeding 40% indicates effective treatment. Additionally, except for the Lipiodol + TRRP@DOX group, varying degrees of pulmonary tumor metastasis were observed in the other three groups, with the control group exhibiting the most severe metastasis and the Lipiodol + DOX group the least.

The occurrence of pulmonary tumor metastasis in the Lipiodol + DOX group may stem from the iodized oil DOX emulsion’s instability, resulting in rapid drug release. Conversely, TRRP@DOX’s slow release profile maintains high local drug concentrations, effectively inhibiting tumor proliferation and systemic metastasis. This study’s approach mimics the clinical TACE strategy, combining TRRP@DOX with iodized oil, overcoming potential embolization limitations due to TRRP@DOX’s small particle size. Through comparison with iodized oil + DOX, this study underscores TRRP@DOX’s enhanced efficacy and reduced systemic toxicity, highlighting its promise in liver cancer treatment.

## Conclusions

TRRP exhibits excellent biocompatibility and forms TRRP@DOX nanoparticles with DOX, allowing targeted uptake by liver cancer cells and controlled release of DOX under intracellular reducing conditions. TRRP@DOX effectively inhibits tumor growth compared to DOX alone while reducing systemic toxicity. This study introduces a promising drug delivery strategy for clinical TACE treatment of liver cancer, with significant implications for future research.

## Supplementary Information

Below is the link to the electronic supplementary material.

Supplementary file1 (DOCX 922 KB)

## Data Availability

The datasets generated during and analysed during the current study are available from the corresponding author on reasonable request.
