# Supplementary material for: Targeted redox-responsive peptide for arterial chemoembolization therapy of orthotropic hepatocellular carcinoma
Source: Abdom Radiol (NY). 2024 Jul 11;49(11):3925–34. doi: 10.1007/s00261-024-04481-8 (PMC11519146; doi:10.1007/s00261-024-04481-8)
Supplement: Supplementary file 1 — Supplementary file1 (DOCX 922 KB) [file 261_2024_4481_MOESM1_ESM.docx]

**Targeted Redox-Responsive Peptide for arterial chemoembolization therapy of orthotropic hepatocellular carcinoma**

Yimao Xia^1^, Xin Li^2^  Fengyong Liu^1，2，*^

1 Chinese PLA Medical School, Beijing 100853, China.

2 Department of Interventional Radiology, The Fifth Medical Center of PLA General Hospital, Beijing 100039, China.

*Corresponding author: Fengyong Liu^1，2，*^

E-mail: liufengyong301@163.com（Fengyong Liu^1，2，*^）

**Materials and methods**

**1 Synthesis of TRRP**

TRRP was synthesized using manual peptide synthesis with fmoc chemistry on 2-Chlorotrityl Chloride resin (1.1 mmol/g capacity). Initially, Fmoc-His(Trt)-OH (15 mmol) was coupled to 2-CTC (5 mmol) resin in situ with N,N-Diisopropylethylamine (3 mL, 15 mmol) in N,N-Dimethylformamide (DMF). After Fmoc deprotection using 20% piperidine in DMF, Fmoc-Lys(Fmoc)-OH (9.65 g, 15 mmol) was coupled to the amino group of serine using N,N'-Diisopropylcarbodiimide (3.5 mL, 22.5 mmol) and 1-Hydroxybenzotriazole (3.05 g, 22.5 mmol) in DMF for 2 hours. The peptide synthesis continued by repeating the Fmoc deprotection and coupling steps based on the peptide sequence, starting from the C terminus and moving towards the N terminus.

To release the peptide from the resin, the fully protected peptide resin was stirred in a mixture of trifluoroacetic acid: 1,2-ethanedithiol: H2O: Trisopropylsilane (94:2.5:2.5:1, v:v) at room temperature for 2 hours. The resulting cleavage mixture was filtered to remove the resin, and the filtrate was precipitated and washed four times with ice-cold diethyl ether. The crude peptide was then purified using semi-preparative Reversed-phase high-performance liquid chromatography (RP-HPLC), followed by lyophilization. The purified peptide was characterized using analytical RP-HPLC and Electrospray ionization-Mass spectrometry (ESI-MS).

**2 Preparation and Characterization of the TRRP@DOX**

Preparation of TRRP@DOX Micelles using dialysis method. First, 10 mg of DOX and 50 mg of the TRRP were separately dissolved in 10 mL of DMF and 10 mL of deionized water, respectively. Under stirring conditions, the TRRP aqueous solution was slowly added dropwise to the DMF solution of DOX while avoiding light, and the reaction was allowed to proceed for 24 hours to ensure sufficient mixing of the TRRP and DOX, resulting in the formation of micelles. Subsequently, the micelles were transferred to a dialysis bag (MWCO = 1000 Da) and dialyzed in deionized water for 24 hours with periodic changes of the water to remove DMF and free DOX and TRRP from the mixed solution. Finally, the micelle solution was frozen rapidly using liquid nitrogen and subjected to freeze-drying. The obtained solid powder was weighed and stored in a light-protected manner.

The morphologies of the TRRP@DOX were observed using a Hitachi HT700 transmission electron microscope (TEM). The Fourier transform infrared (FTIR) spectra and fluorescence spectrum of TRRP, DOX, and TRRP@DOX were recorded using a FTIR Spectrometer (Nicolet 6700 Thermo fisher) and Fluorescence Spectrophotometer (F2700 HITACHI), respectively. The UV–Vis–NIR spectrum and dynamic light scattering (DLS) size distribution of TRRP@DOX were measured by using an UV–Vis spectrometer (UV-2550 Shimadzu) and a Malvern Zetasizer (Nano ZS90 Malvern), respectively.

DOX was quantified using UV-vis at a wavelength of 480 nm to calculate the drug loading content (DLC) and efficiency (DLE) according to the following equations:

$$DLC\left( wt\%. \right)=\frac{weight of loaded DOX}{total weight of polymer and loaded DOX}\times100$$

$$DLE\left（ \% \right）=\frac{weight of loaded DOX}{weight of DOX in feed}\times100$$

**3 DOX release behavior**

The release of DOX in TRRP@DOX was determined by dialysis method. Briefly, TRRP@DOX were put into the dialysis bag (1000 KD) and separately put into GSH concentration of 8 mM (tumor cells cytosolic concentration), 0.2 mM (normal cells cytosolic concentration) and 2 µM (plasma concentration) at 37 ℃. The release solution was taken 2 mL at the indicated time point with suppled 2 mL fresh GSH solution. The DOX content was further analyzed on their absorbance spectrum at 480 nm.

**Result**


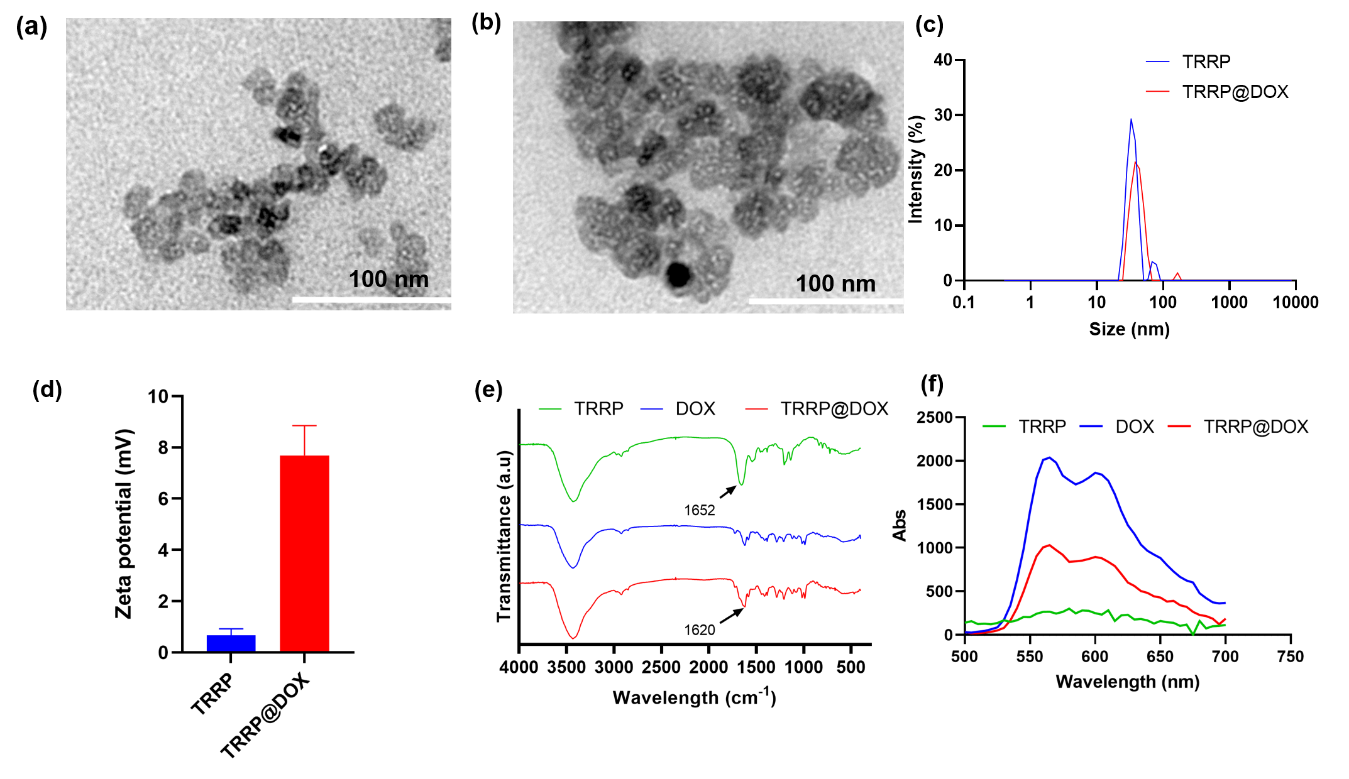


**Figure S1.** Characterization of TRRP and TRRP@DOX. (a) TEM images of TRRP. (b) TEM images of TRRP@DOX. (c) DLS measurement of TRRP and TRRP@DOX. (d) Zeta potential of TRRP and TRRP@DOX. (e) FT-IR spectra of TRRP, DOX, and TRRP@DOX. (f) Emission spectra of TRRP, DOX, and TRRP@DOX at different excitation wavelengths.


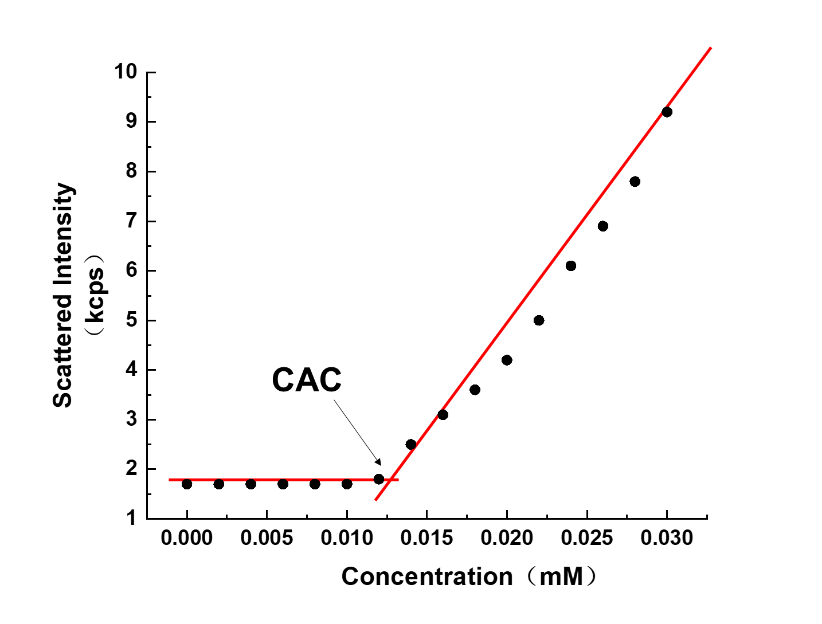


**Figure S2**. Scattered intensities collected from the different concentration solutions of TRRP in water. The intersection point in the plot corresponds to CAC.


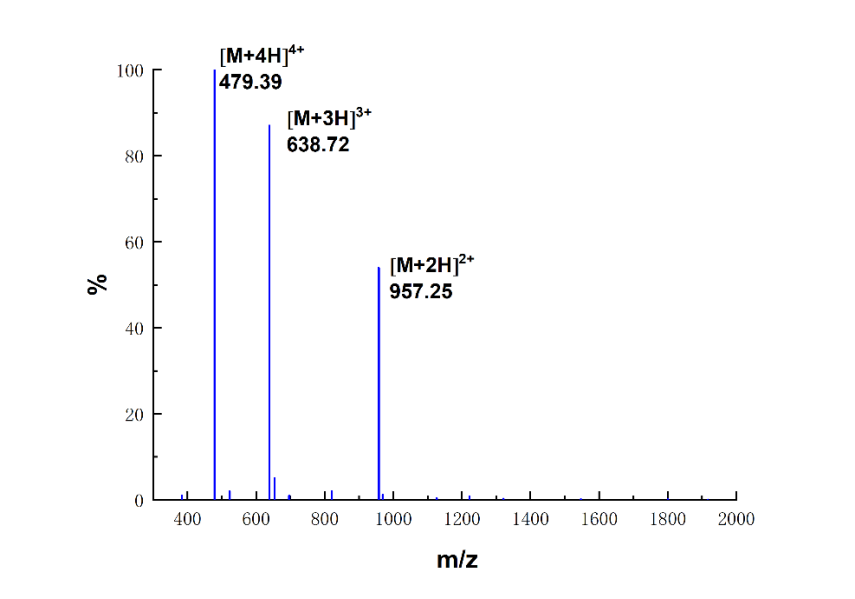


**Figure S3**: ESI-MS spectra of TRRP

The synthesized TRRP (CWK(WC)GGGGSHAIYPRH) comprises two modules: a targeting module and a drug-carrying module. Leveraging prior research indicating overexpression of TfR on liver cancer cells, we employed the T7 peptide, recognized for its TfR-targeting capability, as the targeting module. The drug-carrying module, composed of cysteine and tryptophan, is linked to the targeting module via a flexible linker. Importantly, a disulfide bond serves as a switch for redox-triggered drug release. Additionally, the indole ring on the tryptophan side chain facilitates π-π interactions, contributing to the self-assembly properties of TRRP. TEM images (Figure.S1 a, b) illustrate that both TRRP and TRRP@DOX exhibit well-defined spherical morphology, with TRRP@DOX micelles showing a well-monodispersed size distribution, averaging around 35.8 nm, larger than TRRP alone (about 24.2 nm). DLS measurements indicate average sizes of approximately 33.2 nm for TRRP and 43.4 nm for TRRP@DOX (Figure.S1c), meeting the size requirements for the enhanced permeability and retention effect. Zeta potentials of TRRP and TRRP@DOX were measured at 0.662 mV and 7.3 mV (FigureS1.d), respectively. The critical aggregation concentration (CAC) of TRRP in water was found to be 1.21 × 10-2 mM (Figure.S2). ESI-MS was utilized to characterize the structure and composition of all samples, confirming the desired product based on significant absorption peaks. Additionally, infrared spectroscopy verified the ordered structures. TRRP displays a characteristic peak at 1652 cm^-1^, suggesting an α-helix secondary structure. Upon co-assembly with DOX, characteristic peaks of DOX are observed at 1284 cm^-1^ and 1384 cm^-1^, confirming successful doxorubicin encapsulation. Moreover, the characteristic peak at 1652 cm^-1^ shifts to 1620 cm^-1^ due to conjugation effects, indicating TRRP's secondary structure transformation from α-helix to β-sheet upon DOX addition (Figure.1e).

Drug loading capacity (% LC) and drug encapsulation efficiency (% EE) of TRRP were estimated via the standard UV-vis calibration curve. The LC and EE were calculated at 29.07% and 41.43%, respectively, comparable to other reported redox-responsive drug carriers.


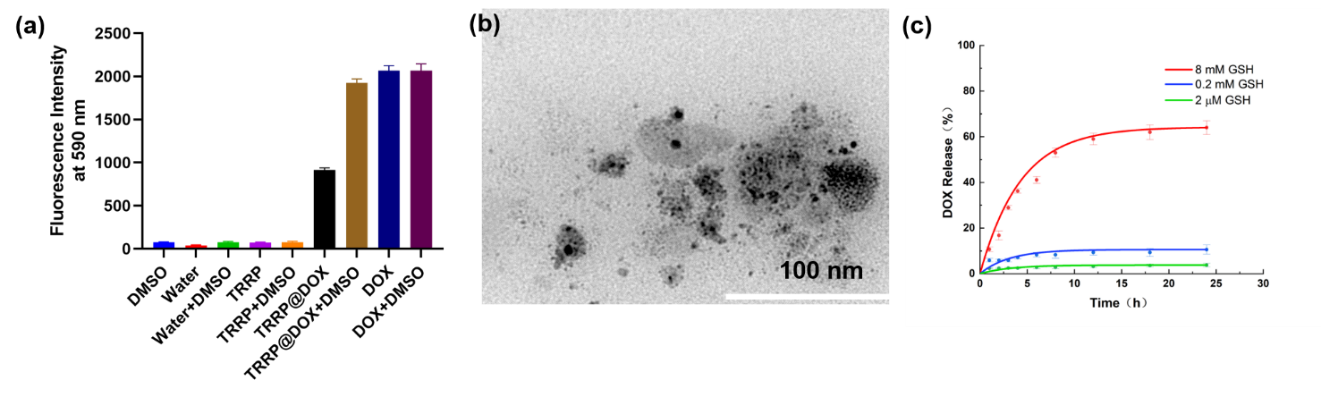


**Figure S4.** In vitro evaluations of redox-responsive drug release of TRRP@DOX. (a) Histogram showing the DOX fluorescence intensity distribution at 590 nm. (b) TEM images of TRRP treated by GSH. (c)Morphological analysis of TRRP after the addition of GSH(8mM). Cumulative drug release of DOX in different concentrations of GSH (8 mm，0.2 mm，0.1 mm, respectively).

**Figure S3**: Ultraviolet standard curve of DOX Y=0.0055X-0.0039（R^2^=0.9662）

To confirm the successful encapsulation of DOX within TRRP micelles, fluorescence spectroscopy analysis was conducted on DOX-loaded micelles (Figure S1.f). DOX typically emits fluorescence at 590 nm; however, this fluorescence can be quenched when confined within a limited volume. As shown in Figure S2.a, drug-loaded micelles (TRRP@DOX) exhibited minimal fluorescence emission, indicating the self-quenching effect on encapsulated DOX fluorescence. Interestingly, a sharp increase in fluorescence intensity was observed upon the introduction of DMSO to DOX-loaded TRRP micelles (TRRP@DOX+DMSO). In polar solvents like DMSO, the hydrogen bonding interactions among peptide bonds are weakened by solvent molecules, leading to micelle disruption and drug release.Furthermore, it is crucial to evaluate the specific GSH reactivity of TRRP micelles to ensure their ability to undergo cleavage and release drugs within tumor cells. TRRP micelles were pretreated with 8 mM GSH to simulate the tumor intracellular environment in vitro, and the GSH-responsive performance of TRRP micelles was assessed by TEM. TEM images revealed that the spherical structure of TRRP, after treatment with GSH, was disrupted and lost its normal morphology (Figure S2.b). The redox-responsive behavior of DOX-loaded micelles and their in vitro DOX release kinetics were systematically investigated across a spectrum of GSH concentrations. At plasma GSH levels of 2 µM and cytosolic GSH concentrations similar to those in normal cells (0.2 mM), the drug release profile remained low, constituting less than 9% of the encapsulated payload. In contrast, exposure to the elevated cytosolic GSH concentration of tumor cells (8 mM) resulted in a maximum DOX release of 64% after 24 hours (Figure S2.c). This observation provides compelling evidence for the precise and controlled DOX release dynamics orchestrated by TRRP micelles within the intracellular milieu, preventing premature drug liberation. The release of DOX from TRRP was relatively slower compared to most reported redox-responsive polymeric systems, suggesting that TRRP has the potential to serve as sustained delivery vehicles.
